# Sodium valproate-induced nocturnal enuresis in epilepsy: Three case reports and a review of the literature

**DOI:** 10.3389/fneur.2023.1104251

**Published:** 2023-03-14

**Authors:** Xiaorong Zhang, Wei Lu, Xiaoye Mo

**Affiliations:** ^1^Department of Neuroendocrinology, The First Veterans Hospital of Sichuan Province, Sichuan Province Revolutionary Disable Armyman Rehab, Chengdu, China; ^2^Department of Neurology, Second Xiangya Hospital, Central South University, Changsha, China; ^3^Department of Emergency, Xiangya Hospital, Central South University, Changsha, China

**Keywords:** sodium valproate, enuresis, side effect, literature review, case report

## Abstract

**Background:**

Enuresis is an uncommon adverse effect of sodium valproate therapy that is unknown to most clinicians. This study provides an overview of the literature on enuresis associated with sodium valproate therapy, discussing the clinical manifestations and possible mechanisms of this side effect.

**Methods:**

We reported three cases of enuresis induced by sodium valproate and reviewed the published enuresis cases associated with sodium valproate therapy retrieved from databases.

**Case presentation and results:**

Three new patients with epilepsy who presented with enuresis following sodium valproate therapy were reported, and 55 published cases of nocturnal enuresis associated with sodium valproate were evaluated. The average age of these patients varied from 4 to 20 years. A total of 48 cases had generalized seizures, seven had focal seizures, and three had unknown seizures. In all the patients, the plasma concentration of sodium valproate was 80.76 ± 14.80 μg/mL, within the therapeutic range when enuresis occurred. With discontinuation or reduction of the drug, all the patients recovered completely.

**Conclusion:**

Sodium valproate-induced enuresis is a rare and reversible side effect, occurring at a younger age, characterized by the generalized onset of seizures, and a rather high dose. The possible mechanisms include insufficient secretion of anti-diuretic hormones, sleep disorder, and hyperactivity of the parasympathetic system. Clinicians should be aware of this uncommon side effect to avoid an incorrect adjustment of the treatment direction.

## Background

Sodium valproate (VPA) is an effective drug in the treatment of epilepsy in adults and children, as well as migraine, bipolar disorder, and trigeminal neuralgia (1). As a kind of GABAergic agonist, VPA enhances GABAergic activity in the brain, which subsequently controls seizures (2, 3). Most side effects of VPA are mild and transient, including vomiting, nausea, abdominal pain, headache, tremor, and drowsiness (3, 4). However, several adverse events induced by VPA, such as amnesia, hair loss, increased body weight, pancreatitis, hyperammonemia in the blood, and nocturnal enuresis, are comparatively infrequent and recognized by clinicians, which may be detrimental to treatment adherence (4–7).

Nocturnal enuresis following treatment with VPA occurs rarely. Statistics on the prevalence of enuresis vary widely from 2 to 24% (1, 8, 9). This unwanted effect is easily overlooked as resulting from the seizures themselves, which might mislead to an erroneous decision to increase the dose of VPA. Moreover, the misdiagnosis of enuresis could make the patient exhibit more psychological symptoms, such as shame, an inferiority complex, timidity, and isolation (10, 11). However, most studies related to VPA-induced enuresis are quite old, and we could not find a recent and comprehensive review of its characteristics and underlying mechanisms.

In this study, we present three unpublished cases of patients with epilepsy who presented with VPA-induced enuresis. In addition, our literature search identified 55 patients with epilepsy who experienced the induction of enuresis after VPA treatment. Based on these examples, we identify the clinical characteristics of VPA-induced enuresis and outline its potential causes.

## Methods

Three new cases of nocturnal enuresis associated with VPA therapy from the clinics of the Second Xiangya Hospital were reported. The English-language literature in the PubMed database was searched from its inception to 30 July 2022, and the Chinese-language literature was searched in Wanfang and CNKI databases (9, 12–17). The following keywords and associated medical subject headings were searched: sodium valproate, enuresis, drug-induced enuresis, and epilepsy. Articles were primarily evaluated for reports on patients diagnosed with epilepsy who suffered from nocturnal enuresis after VPA therapy. We excluded the cases if the diagnosed patients fulfilled at least one of the following criteria: patients who were not epileptic, who lacked the required clinical data, or who had lost the laboratory examination data. Clinical charts, such as age, sex, and types of epilepsy, were reviewed, and the data on drug dosage, time of side effect onset, plasma drug concentration, and prognosis with remission were summarized.

## Case presentation and results

### Clinical data of three new case reports

Patient 1. A 20-year-old woman had generalized tonic-clonic epilepsy since the age of 16 years, with a frequency of one or two times every month. She had no complaints of polyuria, polydipsia, or thirstiness. She had never received any antiepileptic drug before. There was no family history of epilepsy, migraine, or neurological disease. Physical examination showed that her weight was 50 kg, and her height was 162 cm. She had good growth and intelligence. There was no developmental retardation and no abnormal urination. The respiratory, cardiovascular, and digestive systems were normal. The laboratory tests showed a regular blood routine and normal liver and kidney functions. The brain magnetic resonance imaging (MRI) scan was normal. The electroencephalogram (EEG) began with a spike wave rhythm, then the amplitude progressively grew, and the frequency gradually decreased. In the clonic phase, 2.5–6 Hz slow waves began to be inserted, progressively diffused, and the background activity gradually returned. The patient was diagnosed with generalized tonic-clonic epilepsy and started treatment with 200 mg of VPA three times a day (12 mg/kg/d). Unexpectedly, this patient with epilepsy came back for consultation 10 days later because she began to experience enuresis every night from the third day of the treatment. The early morning plasma concentration of VPA was detected at a therapeutic level of 80.2 μg/mL. At that time, we misinterpreted her enuresis as a clinical symptom of epileptic seizures when the patient fell asleep. To strengthen the control of seizures, we recommended increasing her dosage of VPA to 300 mg three times a day (18 mg/kg/d). After 1 month, the patient returned to the clinic and complained of a striking remission in seizures but still had enuresis every night. Then, we wondered whether the enuresis was induced by the start of VPA therapy. Based on our suspicions, the patient gradually reduced VPA and switched to carbamazepine (CBZ) for controlling seizures. A remission of nocturnal enuresis was achieved on the second day of her stopping VPA (Table 1). Afterward, the enuresis did not recur after the discontinuation of VPA for 3 months.

**Table 1:** Clinical data of three unpublished case reports.

**Patient**	**1**	**2**	**3**
Sex	Female	Male	Female
Age(y)	20	9	15
Type of epilepsy	Generalized onset: tonic-clonic	Generalized onset: clonic	Generalized onset: absence
Familial History of Epilepsy	No	No	Yes
Drug formulation	Regular type	Sustained-release type	Sustained-release type
Drug dosage	200 mg TID (12 mg/kg/d)	500 mg QN (18 mg/kg/d)	500 mg QN (11 mg/kg/d)
Accompanied symptoms	None	Urinary incontinence during daytime	None
Time of enuresis occurrence (d)	3	2	1
VPA concentration with enuresis (μg/mL)	80	93.1	72.3
Measures	Discontinuation	Discontinuation	Reduce the dosage to 250 mg QN
VPA concentration with enuresis remission (μg/mL)	N/A	60.8	39.1
Time of enuresis remission (d)	2nd day after discontinuation	1st day after discontinuation	2nd day after discontinuation

CBZ, carbamazepine; VPA, sodium valproate; TID, ter in die, three times a day; QN, quaque nocte, once a night; N/A, not available.

Patient 2. A 9-year-old boy had generalized clonic seizures two times a week for more than 2 months. He suffered from clonic seizures, with the symptoms of muscle contractions alternating with periods of muscle relaxation associated with the loss of consciousness and typically lasting for 30–60 s. There was no familial history of epilepsy or other neurological diseases. The physical examination of the patient showed that his weight was 24.8 kg and his height was 132 cm. His respiratory, cardiovascular, and digestive systems were normal. There was no abnormality in the liver or kidney function. An MRI scan of the brain showed sclerosis in the region of the hippocampus. EEG was characterized by rhythmic 4–6 Hz spike and slow waves in the bilateral frontal lobes. This young boy had started taking CBZ 100 mg three times daily prescribed elsewhere, with which his seizures were not well-controlled. After he visited our clinic, treatment with VPA of 150 mg three times daily (18 mg/kg/d) was initiated, replacing the use of CBZ. After 1 month, the boy was brought for a return visit, complaining of nocturnal enuresis from the first night of VPA treatment, accompanied by urinary incontinence during the daytime. Meanwhile, he developed polyuria and thirstiness, the 24-h urine volume of which was 1,890 mL. His morning predose concentration of VPA was detected at 93.1 μg/mL. Considering his accompanying symptoms of polyuria and thirstiness, we measured his plasma anti-diuretic hormone (ADH) concentration after 12 h of fasting at 8 am, which was at a relatively low concentration of 0.68 pg/mL (normal range, 1–5 pg/mL). Electrolytes in serum, urine-specific gravity, and urine volume were all within the normal range. We carefully asked about his case history. The patient had regular urination behavior before, and his epilepsy was well-controlled with VPA. We suspected that the occurrence of enuresis was due to the relatively low level of ADH caused by the use of VPA. Nocturnal enuresis disappeared 2 days after reducing the dosage of VPA to 200 mg two times daily (16 mg/kg/d). Then, the early morning plasma concentration of VPA decreased to 60.8 μg/mL (Table 1). The subsequent detection of the serum ADH level was rejected by his parents. Enuresis did not recur after his reduction in the dosage of VPA.

Patient 3. This was a 15-year-old boy who complained of the generalized absence of seizures for 2 years. His absence of seizure was characterized by an abrupt cessation of ongoing activities with the loss of consciousness, followed by an abrupt return to his former normal behavior. There were no accompanying symptoms of polyuria, polydipsia, or thirstiness. The frequency of his seizures was about three or four times a month. There was a strong family history of epilepsy; his mother had tonic-clonic seizures 20 years ago. He had never taken an antiepileptic drug before. Physical examination showed a weight of 45 kg and a height of 155 cm. He had no mental or developmental retardation. He had fairly normal growth and intelligence. Urination was normal. The respiratory, cardiovascular, and digestive systems were normal. The laboratory tests showed a regular blood routine and normal liver and kidney functions. The MRI scan of the brain was regular, while the EEG exhibited a 3–5 Hz spike and slow waves. Treatment with sustained-release VPA (Depakine Chrono) of 500 mg every night (11 mg/kg/d) was initiated after he visited our clinic. The boy was brought back to the clinic by his father 7 days later because enuresis occurred from the first night of Depakine treatment. His plasma concentration of VPA before the morning dosage was 72.3 μg/mL. VPA-induced enuresis was our primary clinical consideration based on our prior experience. A reduction in VPA to 250 mg every night was suggested, and, as expected, a rapid remission of enuresis was achieved 3 days later. The plasma concentration was examined again at the remission time of enuresis, showing a decreased level of 39.1 μg/mL (Table 1). Unfortunately, the boy had a recurrence of seizures, so he initiated taking lamotrigine for controlling seizures afterward.

### Clinical data of published cases

A total of eight related studies were retrieved with terms in both English and Chinese. These studies included 55 detailed cases of VPA-induced enuresis meeting the criteria. In total, two cases were reported in Chinese, and the remaining 53 cases were in English. Table 2 lists the clinical characteristics of these 55 published cases of VPA-induced enuresis in patients with epilepsy, including age, sex, type of epilepsy, therapeutic drug dosage, plasma VPA concentration, the time of enuresis occurrence, and prognosis.

**Table 2:** Clinical data of 55 published cases.

**Authors**	**Patients**	**Age (y)**	**Sex**	**Type of epilepsy**	**Time of enuresis occurrence (d)**	**VPA concentration with enuresis (μg/mL)**	**Drug dosage (mg/kg/d)**
Fan et al. (12)	1	6	F	Focal impaired awareness	42	N/A	30-40
	2	8	M	Focal impaired awareness	60	N/A	30–40
Shi (13)	3	4	F	Focal awareness	1	55	100 mg TID
Panayiotopoulos (14)	4	6	F	Generalized onset non-motor	2	52.2	200 mg TID
	5	6	F	GTC	3	60	19
Esmael et al. (15)	6	5	M	GTC	5	65	26
	7	5	M	Generalized onset non-motor	6	59	26
	8	5	F	GTC	11	67	24
	9	5	M	GTC	23	96	32
	10	5	M	GTC	20	89	36
	11	5	F	Generalized onset motor (myoclonic)	25	96	30
	12	6	F	Generalized onset non-motor (absence)	12	91	36
	13	6	M	GTC	15	73	24
	14	6	M	GTC	16	85	26
	15	6	M	GTC	14	71	23
	16	6	F	GTC	23	81	24
	17	7	F	Focal onset	23	90	31
	18	7	M	GTC	10	85	31
	19	7	M	GTC	18	85	25
	20	7	F	GTC	19	92	30
	21	8	M	Focal onset	9	94	35
	22	8	F	Generalized onset motor (myoclonic)	35	81	22
	23	8	F	Focal onset	17	87	29
	24	8	M	GTC	14	88	32
	25	9	M	GTC	28	84	33
	26	9	M	GTC	19	87	30
	27	9	F	Focal onset	20	85	29
	28	10	F	Unknown onset	9	87	30
	29	10	M	GTC	35	92	32
	30	11	F	GTC	15	90	31
	31	11	M	GTC	35	95	31
	32	12	M	Generalized onset motor (myoclonic)	17	89	34
	33	12	F	GTC	33	91	35
Ozan et al. (9)	34	5	F	GTC	4	55	25
	35	5	M	GTC	5	55	25
	36	6	M	GTC	10	64	23
	37	6	M	GTC	22	45	38
	38	6	M	GTC	19	67	37
	39	7	F	GTC	24	56	25
	40	7	F	GTC	11	77	22
	41	7	M	GTC	14	74	23
	42	7	M	GTC	15	91	30
	43	8	M	GTC	13	82	32
	44	9	M	GTC	22	77	28
	45	9	M	GTC	9	97	30
	46	9	F	Generalized onset non-motor (absence)	17	82	34
	47	10	M	Generalized onset tonic	18	73	21
	48	11	M	Generalized onset motor (myoclonic)	8	89	32
	49	11	M	GTC	34	97	35
	50	11	F	Generalized onset motor (myoclonic)	14	98	35
	51	12	F	GTC	34	86	34
	52	12	F	Generalized onset tonic	16	78	32
	53	13	M	Generalized onset non-motor (absence)	32	87	35
Gosavi et al. (16)	54	5	M	Unknown onset	30	N/A	25
Zaïem et al. (17)	55	11	M	Febrile seizure	2	124	500 mg BID

GTC, generalized onset motor (tonic-clonic); VPA, sodium valproate; TID, ter in die, three times a day; BID, bis in die, two times a day; N/A, not available.

### Combined analysis of all the presented cases

In total, 58 patients with epilepsy (33 M, 25 F) between the age of 4 and 20 years were analyzed. All the patients had normal urination before VPA therapy. None of them had delayed development, fecal incontinence, mental disorder, or intellectual disturbance. Regarding the type of epilepsy, 49 of them had generalized seizures (82.76%), seven had focal seizures (12.07%), and three had unknown seizures (5.17%). The days of enuresis onset was 17.47 ± 11.68 days after VPA treatment. The VPA dosing for the reviewed cases was 28.45 ± 5.98 mg/kg/d. The plasma VPA concentrations varied from 52.2 to 124.0 μg/mL when enuresis occurred (with a therapeutic range from 50 to 100 μg/mL) though the drug concentration was not measured for three of them. Only one of the patients had accompanied symptoms of urinary incontinence during the daytime. Of interest, all the presented patients had satisfactory prognoses that enuresis disappeared completely after reducing or stopping VPA (Table 3).

**Table 3:** Analysis of all the presented cases.

**Parameters**	**Cases (percentage)**
Sex (M: F)	33:25
Age (y)	8.18 ± 2.96
**Clinical types of epilepsy**	
Generalized onset	48 (82.76%)
Focal onset	7 (12.07%)
Unknown Onset	3 (5.17%)
Time of enuresis occurrence (d)	17.47 ± 11.68
Plasma VPA concentration (μg/mL)	80.76 ± 14.80
Drug dosage (mg/kg/d)	28.45 ± 5.98
**Prognosis**	
Satisfactory	58 (100%)
Unsatisfactory	0 (0%)

VPA, sodium valproate; M, male; F, female.

## Discussion and conclusion

In this study, we described three new cases of patients with epilepsy with VPA-induced enuresis. Combining these with 55 published reported cases, we summarized that VPA-induced nocturnal enuresis occurred not only in children but also in young adults. The likelihood of this side effect may be correlated with younger age, a generalized onset of epilepsy, and a relatively high drug dose. Gender, plasma concentration, and duration of treatment do not appear to be the risk factors.

Nocturnal enuresis is a condition of intermittent urinary incontinence that occurs during periods of sleep; it is divided into primary and secondary types (10). Most cases of enuresis occurring on VPA treatment are secondary (17). As an adverse effect, secondary enuresis was previously reported only in pediatric patients, and there has been no literature examining adult patients, which may be attributed to two factors. First, younger age was reported to be the only significant factor in predicting the development of enuresis (1, 15). To our knowledge, in this study, we reported the first case of VPA-induced enuresis in an adult patient with epilepsy. The common causes of nocturnal enuresis in adults include urinary disease, the use of certain drugs, sleep disorders, mental status, and metabolic disorders (10, 18). Unfortunately, we did not evaluate the patient for urinary tract infection, genitourinary anatomical abnormalities, or sleep quality in this study. Second, patients rarely spontaneously report this symptom of enuresis. Yamak et.al. found that only 4% of children had enuresis spontaneously reported by their parents and that the percentage of adult patients who spontaneously reported enuresis was < 4% (1). This emphasizes the importance of neurologists specifically inquiring about nocturnal enuresis since this adverse event is rarely spontaneously reported.

In our study, we stated that the average VPA concentration of all the 58 presented cases at the onset of enuresis was 80.76 μg/mL, which was within the recommended effective therapeutic range. Similarly, Esmael reported that, compared to the non-enuresis group, VPA concentration in the enuresis group was higher, ranging from 75.44 to 94.2 μg/mL (15). Ozan et al. also reported that the VPA concentration was 76.5 ± 3.4 μg/mL when enuresis occurred, which was within the therapeutic range, while in the non-enuresis group, it was 70.8 ± 16.9 μg/mL (9). The efficient therapeutic concentration range of VPA for controlling seizures varies from 50 to 100 μg/mL. Together, enuresis is a side effect that can occur at drug concentrations in the therapeutic range. Regarding drug dosage, the recommended therapeutic dosage is 3–40 mg/kg/d. In our study, 58 presented patients were given a recommended dosage, but at least 50 of them received doses of more than 20 mg/kg/d. All of them ceased to experience enuresis after stopping or reducing the drug. This was similar to the findings of Cheng et al. (19). The findings indicated that when the dosage is relatively larger, this reversible side effect is more inclined to occur.

How VPA induces nocturnal enuresis remains unclear, although several mechanisms have been emphasized. ADH is synthesized and secreted in the hypothalamic paraventricular nucleus and acts as a potent vasoconstrictor and anti-diuretic hormone (18, 20). The insufficient secretion of ADH plays a role in the pathophysiology of child enuresis, and ADH replacement therapy was effective in treating some children with enuresis (20, 21). Previous clinical studies have reported that VPA can induce an insufficient secretion of ADH, which could impair renal water excretion and non-osmolar stimulation. Specifically, VPA, as a GABAergic agonist, is capable of inhibiting the increase in ADH in response to physical exercise and smoking (22, 23). Coir et al. stated that VPA could significantly inhibit the angiotensin II-induced increase in ADH (24). In our study, due to the accompanying symptoms, Patient 2 evaluated his serum ADH level in the early morning, which was at a lower level than normal. It seems that VPA-induced enuresis is related to its inhibition of ADH secretion. Unfortunately, we did not further test the patient's urine-plasma osmotic pressure ratio and ADH levels after enuresis resolution.

Another proposed mechanism is that VPA increases the deep sleep period and slow-wave sleep activity. Deeper sleep and increased slow brain-wave activity are potential reasons for enuresis (9). Esmael et al. carried out polysomnography for 28 children with secondary VPA-induced enuresis and 28 age- and sex-matched control children (15). They found that latency, the number of awakenings, sleep arousal, and the snoring index were significantly increased, and the total sleep time, sleep efficiency, rapid eye movement sleep percentage, and lowest oxygen saturation were significantly reduced (15, 25). VPA has been reported to affect sleep by mediating melatonin (26, 27). Melatonin activation caused by VPA induced deeper sleep and an increased sleep need, thus leading to an impaired ability to awaken to the sensation of bladder contractions and more frequent urination (9, 28). However, we did not perform polysomnography in our hospital. Moreover, increased parasympathetic activity, a change in the renal tube, and a central effect on the thirst center were considered three other possible reasons for enuresis (9, 29–31). More large-scale clinical studies to confirm the roles of ADH level, sleep monitoring, parasympathetic activity, and renal tube function are needed to elucidate the development of VPA-induced enuresis.

Nocturnal enuresis, though not a life-threatening adverse event, is an underreported and overlooked symptom caused by VPA. It may occur in children and young people. Although the mechanism of VPA-induced enuresis is unclear, it is defined as a functional and reversible disorder characterized by easy onset at a younger age, a generalized onset of seizures, and a rather high dose. There are some limitations to this study. The unpublished sample size was too small. Most of the published patient data lacked important information and the performance of polysomnography. In addition, sleep disturbance does not explain enuresis in those patients with a concurrent daytime frequency of urination. In conclusion, neurologists should be aware of this adverse effect and proactively inquire about this symptom from patients or family members. Nocturnal enuresis can be misinterpreted as resulting from an epileptic attack, which might lead to an erroneous clinical decision.

## Data availability statement

The original contributions presented in the study are included in the article/supplementary material, further inquiries can be directed to the corresponding author.

## Ethics statement

The studies involving human participants were reviewed and approved by the Ethics Committee of the Second Xiangya Hospital of Central South University. Written informed consent to participate in this study was provided by the participants' legal guardian/next of kin. Written informed consent was obtained from the individual(s) and/or minor(s)' legal guardian/next of kin for the publication of any potentially identifiable images or data included in this article.

## Author contributions

XZ wrote the manuscript. XM was involved in draft correction and editing. WL and XM were involved in the clinical management of patients and data collection. All authors read and approved the final manuscript.
